# Self-Perceived Health, Comorbidity, and Burden Among Older Family Caregivers of Seniors with Severe Mental Disorders: A Cross-Sectional Study

**DOI:** 10.3390/ijerph23050544

**Published:** 2026-04-22

**Authors:** Ana Carolina Gama, Claudia Marcela Chimbí, Margarita María Benito Cuadrado, Jose Manuel Santacruz Escudero, Cecilia de Santacruz, Diego Andrés Chavarro-Carvajal

**Affiliations:** 1 Instituto de Envejecimiento de la Facultad de Medicina, Pontificia Universidad Javeriana, Bogotá 110231, Colombia; j.santacruz@javeriana.edu.co (J.M.S.E.); me000693@javeriana.edu.co (C.d.S.); chavarro-d@javeriana.edu.co (D.A.C.-C.); 2 Intellectus, Centro de memoria y Cognición, Hospital Universitario San Ignacio, Bogotá 110231, Colombia; cchimbi@javeriana.edu.co (C.M.C.); mmbenito@husi.org.co (M.M.B.C.); 3 Departamento de Psiquiatría y Salud Mental de la Facultad de Medicina, Pontificia Universidad Javeriana, Bogotá 110231, Colombia

**Keywords:** self-perceived health, older caregivers, severe mental disorder, caregiver burden, comorbidity

## Abstract

**Highlights:**

**Public health relevance—How does this work relate to a public health issue?**

- Older caregivers of individuals with severe mental disorders represent a growing vulnerable population that may exacerbate existing health inequalities.

**Public health significance—Why is this work of significance to public health?**

- Functional and emotional dimensions show stronger associations with self-perceived health than the number of comorbidities among older caregivers of people with severe mental disorders.

**Public health implications—What are the key implications or messages for practitioners, policy makers and/or researchers in public health?**

- Public health policies aimed at older caregivers must transcend the disease-centered approach, emphasize gender equity, and provide well-being tools in the functional and emotional dimensions.

**Abstract:**

The global aging process has increased the number of older individuals providing care for relatives with severe mental disorders (SMD). This population faces unique health challenges. The present cross-sectional study examined the relationship between self-perceived health (SPH) and clinical, functional, and sociodemographic variables among 71 older caregivers (median age: 65 years) in Bogotá, Colombia. SPH was assessed by answering the question: “How would you describe your overall health status?” and dichotomized into good versus poor perception. Comorbidity was measured as the number of self-reported chronic conditions. Caregiver burden was evaluated using the Zarit Caregiver Burden Interview, and health-related quality of life (HRQoL) was assessed using the SF-36, including dimensions such as physical functioning, emotional well-being, bodily pain, and general health. Descriptive analyses, non-parametric comparisons, and logistic regression models were conducted. The results revealed a marked feminization of caregiving (92.96%) and a high prevalence of good SPH (70.42%), despite a substantial burden of physical comorbidities (mean: 3.21). Dimensions such as physical functioning, emotional well-being, and pain were significant in univariate analyses. However, the multivariate model identified general health as the only independent predictor of good SPH (adjusted odds ratio [OR]: 1.112; 95% confidence interval [CI]: 1.053–1.174; *p* < 0.001). These findings suggest that subjective health assessment may transcend objective disease counts for older caregivers. Public health policies could prioritize wellness-based interventions and emotional support over traditional disease-centered approaches to improve the quality of life of this growing, active, socially valuable, yet vulnerable population.

## 1. Introduction

Population aging is a global phenomenon reshaping social and family structures. Currently, the world population is estimated at 8.083 billion people, of whom approximately 10% are aged 65 years and older [1]. By 2050, the population aged 60 years and over is expected to reach 2.1 billion [2]. In Colombia, this demographic shift is also significant; according to data from DANE, older adults currently account for 15% of the population, and it is projected that by 2070 they will constitute 36% of the national total [2].

The growing proportion of older adults is associated with a higher incidence of chronic diseases, including hypertension, diabetes, and major neurocognitive disorders (MNCD), also known as dementias. Nearly 139 million people worldwide are expected to live with MNCD by 2050 [3]. These pathologies are accompanied by progressive functional decline and long-term care needs, resulting in a significant familial, social, and economic impact [4]—a situation commonly observed in other severe, long-term psychiatric disorders. In Latin America and the Caribbean, care is predominantly provided by families; 60% of this care is delivered by women, who are 8% less likely to have formal employment [5]. In Colombia, there were 6.8 million full-time unpaid caregivers in 2023, 85.7% of whom were women [6]. Among older adults, 1,232,335 live with a disability [7]; 70.7% of those requiring care receive it from a cohabitant, primarily a woman (86%). Notably, 43% of these caregivers are also older adults, and only 5.6% are remunerated [8].

Evidence indicates that while caregiving can improve resilience and a sense of purpose [9], it is also associated with negative effects across physical, emotional, and social domains. Older caregivers often report perceived deterioration in mental health linked to emotional bonds, burden, and lack of support. Due to cohabitation, family caregivers devote substantial time to work (averaging 10 h daily) and maintain a strong emotional relationship that may exhibit characteristics of codependency [10,11].

Furthermore, for caregivers, who are primarily family members and, as mentioned, mostly women, quality of life encompasses multiple elements, such as emotional well-being, interpersonal relationships, personal development, material needs, rights, self-determination, and social inclusion [12], in addition to their own well-being and that of the care recipient. To ensure the well-being of the person in their care, caregivers must modify their daily lives, which can lead to worry, stress, mood changes, constant fear, physical and emotional deterioration, self-neglect, job abandonment, health problems, decompensation of underlying diseases, lack of treatment adherence, social isolation, and poor self-care skills. These caregiving-related demands may contribute to caregiver burden syndrome [13]. In turn, greater caregiver burden has been associated with poorer self-rated health, whereas caregivers who do not perceive themselves as overburdened tend to report better SPH and mental health [14,15,16,17].

Self-Perceived Health (SPH), typically measured through a single question asking individuals to assess their own health status—either in an open-ended format or using a 3- or 5-point response scale—is widely considered a valid subjective indicator of both health status and the factors individuals associate with it. Its value lies in its multidimensional nature, as it integrates physical, psychological, and sociocultural aspects of health into a global personal judgment. SPH is conceptually related to the General Health domain of the SF-36, which ranges from excellent to poor health. In older adults, SRH has been linked to physical functioning, disease burden, disability, and functional limitations, and has also been associated with important outcomes such as mortality, hospitalization, functional decline, and healthcare use [14]. It can also be linked to the notion of quality of life, particularly health-related quality of life (HRQoL), a multidimensional construct focused on the subjective impact of disease or therapeutic interventions on everyday functioning. In this regard, the SF-36 is a self-reported instrument that assesses both perceived health and HRQoL [18,19].

Associations have been found between poor SPH and factors such as age, female gender, unhealthy lifestyle, functional limitations, number of diseases, and poor social support [14,15,16]. However, previous studies in Colombia (SABE Survey) concluded that most caregivers report good SPH despite physical fatigue [20], while a reduction in SPH related to care burden has also been reported [10,14,15].

To date, available scientific evidence on health conditions and SPH among older caregivers of relatives with severe mental disorders in Latin America is limited. It is hypothesized that this population experiences a higher caregiving burden due to restricted autonomy, increased care needs, and disruption of roles, among other alterations in the care recipient. These conditions require special forms of care, causing differential physical and emotional impacts [5,21]. In this context, the primary objective of this study was to explore the association between SPH in older family caregivers and variables such as comorbidity, pain, and care burden to inform the future development of interventions tailored to their specific needs.

## 2. Materials and Methods

### 2.1. Study Design and Population

A cross-sectional analytical study was conducted. The study population consisted of individuals aged 60 years and older who had dedicated at least 24 h per week for more than six months to caring for older relatives with severe mental disorders, defined as mental health conditions associated with substantial functional impairment, including major neurocognitive disorders (dementia) due to their functional impact and high caregiving demands and functional dependency [22] (Barthel Index score < 60 points). A convenience sample was used, comprising 71 participants (*n* = 71) enrolled in a broader study on caregiver burden and self-care interventions. Given the exploratory nature of this study and the specific clinical profile of the population, this sample size was considered sufficient for initial association modeling, although it limits the generalization of the findings.

### 2.2. Procedures and Ethics

The research was conducted in accordance with the principles of the Declaration of Helsinki. The study protocol was submitted for evaluation and received formal approval from the institutional Research Ethics Committee (protocol code 070-2023; approved on 8 June 2023). The principle of autonomy was ensured by obtaining written informed consent from each participant prior to data collection. The custody, confidentiality, and anonymity of the information were strictly guaranteed.

### 2.3. Data Collection and Instrument

Data were collected using standardized and validated instruments to ensure reliability. The following variables and tools were employed:

- Sociodemographic and Clinical Data: Information on age, sex, number of comorbidities, and presence of pain was obtained through self-report questionnaires.
- Self-Perceived Health (SPH): SRH was defined as the primary outcome variable. Participants were asked the question: “*How would you describe your overall health status?*” Because responses were collected in qualitative terms, they were subsequently recoded into two categories based on their semantic and evaluative content. Responses indicating an overall positive perception of health were classified as Good SRH, including *good*, *good health*, *excellent*, *good but has declined*, *good at the physical level but with emotional difficulties*, *acceptable*, and *stable*. Responses indicating a negative, fragile, or deteriorated perception were classified as Poor SRH, including *fair*, *unstable*, *has deteriorated*, *requiring care*, and *poor*.

The dichotomization of SPH into good versus poor categories is consistent with established epidemiological practice in aging research, where this binary distinction has demonstrated robust predictive validity for mortality, functional decline, and health service utilization. This approach enhances clinical interpretability and facilitates the identification of at-risk individuals in public health settings. Additionally, given the sample size of this study (*n* = 71), a binary outcome was deemed more appropriate to ensure adequate statistical power and model stability in the logistic regression analysis, avoiding the risk of overfitting that could arise from a more finely stratified outcome variable.

- Zarit Caregiver Burden Interview: A 22-item version was used to quantify the degree of caregiving burden. Scores were interpreted as: 0–46 (no burden), 47–55 (mild burden), and 56–88 (severe burden) [23,24].
- SF-36 Health Survey (Colombian Spanish version): Used for the multidimensional assessment of Health-Related Quality of Life (HRQoL). It measures dimensions such as physical functioning, role limitations (physical and emotional), energy/fatigue, emotional well-being, social functioning, bodily pain, and general health. Scores range from 0 to 100, with higher values indicating better health status [25,26].

### 2.4. Statistical Analysis

The data analysis was structured into three phases:

- Descriptive Analysis: Quantitative variables (age and scale scores) were described using medians and interquartile ranges (IQR) or means and standard deviations (SD), depending on their distribution. Categorical variables were presented as absolute and relative frequencies.
- Comparative Analysis: Differences between SPH groups (Good vs. Poor) were evaluated using non-parametric tests, specifically the Mann–Whitney U test for median comparison. The significance threshold was set at *p* < 0.05. Additionally, effect sizes were calculated using Cohen’s d to determine the clinical magnitude of the observed differences, with values of 0.2, 0.5, and 0.8 representing small, medium, and large effects, respectively.
- Association Modeling: A logistic regression model was employed to identify predictors of good SPH. Crude Odds Ratios (OR) were calculated for univariate analysis, and adjusted Odds Ratios (aOR) were calculated using a multivariate model to identify independent factors. All estimates are presented with their respective 95% confidence intervals (CI). To ensure the validity of the multivariate model, multicollinearity was assessed using the Variance Inflation Factor (VIF). Model goodness-of-fit was evaluated using the Hosmer–Lemeshow test and Nagelkerke’s pseudo-R^2^ was reported to estimate the proportion of explained variance.

## 3. Results

### 3.1. Sociodemographic and Clinical Characteristics

The study population consisted of 71 caregivers. The median age of the participants was 65 years (IQR: 62–72). A marked feminization of caregiving was observed, with 92.96% (*n* = 66) being women compared to 7.04% (*n* = 5) men. Regarding physical health status, the mean number of comorbidities was 3.21 (SD: 1.90); the most frequent categories were 2, 3, and 5 concurrent diseases, each representing 19.72% of the sample.

In terms of caregiver burden measured by the Zarit scale, the mean score was 48.46 (SD: 14.87). Notably, 52.11% (*n* = 37) of the participants showed no burden, while 33.80% (*n* = 24) exhibited moderate to intense burden levels. The complete characterization of the sample is presented in Table 1.

### 3.2. Comparative Analysis According to Self-Perceived Health (SPH)

When comparing groups based on their health perception, 70.42% (*n* = 50) reported good SPH. Statistically significant differences (*p* < 0.05) were identified in the functional and emotional dimensions of the SF-36 between participants who perceived their health as good and those who perceived it as poor.

Specifically, the Good SPH group presented significantly higher scores in physical functioning (90 vs. 70; *p* = 0.004) and emotional well-being (72 vs. 48; *p* = 0.008), and a notable difference in pain perception, with a median of 89 compared to 45 in the poor perception group (*p* < 0.001). To assess clinical relevance, Cohen’s d was calculated for significant differences, revealing large effect sizes for Bodily Pain (d > 0.8) and moderate-to-large effects for Physical Functioning and Emotional Well-being (Table 2).

### 3.3. Logistic Regression Analysis

In the univariate analysis (Crude OR), all SF-36 dimensions showed a significant association with the probability of reporting Good SPH. For instance, each unit increase in physical functioning increased the likelihood of reporting good health by 1.042 times (*p* = 0.004).

However, in the multivariate (adjusted) model, most variables lost statistical significance, including age, comorbidities, and the Zarit score. The only variable that remained significantly associated with good SPH was “General Health”: for each unit increase in this score, the probability of having Good SPH increased by 1.112 times (Adjusted OR: 1.112; 95% CI: 1.053–1.174; *p* < 0.001). The final model demonstrated an adequate fit (Hosmer–Lemeshow test: *p* > 0.05) and explained a significant portion of the variance (Nagelkerke R^2^ = 0.54). Collinearity diagnostics showed no significant issues, with VIF values ranging from 1.1 to 2.3 for all predictors. (Table 3).

While this association is robust, it is important to note the potential conceptual overlap between the SPH question and the General Health domain of the SF-36, which likely accounted for a large portion of the model’s variance.

## 4. Discussion

This study examined the relationship between self-perceived health (SPH) and clinical, functional, and burden-related variables in a sample of older family caregivers of individuals with severe mental disorders (SMD) in Bogotá, Colombia. The findings reveal a complex interplay between objective health indicators and subjective health appraisal, with important implications for public health policy and clinical practice. The result suggest a processes of adaptation, resilience, and personal gains [27,28], which have been described as protective mechanisms. These mechanisms enable caregivers to maintain emotional balance and a positive assessment of their well-being despite the continuous demands of caregiving [29].

The marked feminization of caregiving observed in this study (92.96% women) is consistent with broader patterns documented in Latin America and globally [9,18,19]. This gender disparity could be related to deeply rooted cultural norms that assign caregiving responsibilities predominantly to women, often at the expense of their own health, economic opportunities, and social participation [20]. These descriptive findings highlight gender equity in caregiving as a relevant policy domain, including recognition and redistribution of care work, access to respite services, and economic support for caregivers.

The prevalence of good SPH (70.42%) in this sample is notably higher than that reported in the SABE Colombia study, which found that only 42.3% of older adults in Colombia rated their health as good or very good [20]. The consistency of these findings with population reports, such as the SABE Colombia Study, suggests that even in the presence of physical and emotional demands, a significant proportion of caregivers maintain a positive global assessment of their health [18]. This underscores the necessity to examine SPH as a multidimensional construct, rather than as a solely biomedical entity. However, while the majority of participants report positive SPH, the experience of mental health caregiving in Colombia is characterized by ambivalent feelings. Research has documented a transformation of identity and lifestyle among family caregivers, with caregiving becoming the central axis of their existence, often under conditions of scarce social support and institutional stigma [30]. This insight is a critical contribution to the field, underscoring the imperative to devise accompaniment strategies that acknowledge this identity shift and provide personalized caregiving tools.

These caregivers typically engage in extended periods of approximately 10 h per day, experiencing a substantial emotional strain that, within the Latin American context, is inadequately addressed by regulatory frameworks and support systems, which fail to safeguard women’s health against the persistent nature of the disease [31]. The prevalence of moderate or intense burden in our study was 33.8%, a figure that is notably lower than the 78.4% reported in previous research involving caregivers of people with dementia [32,33]. This discrepancy should be interpreted with caution, as it may be associated with heterogeneity in the profiles of care recipients, disease trajectories, and the healthcare context. This finding suggests that caregiver burden alone does not fully explain the poor health perception among older adults. It demonstrates how caregivers of individuals with SMDs employ varied coping strategies. In this sense, resilience could emerge as a potential factor that modulates the caregiving experience, transforming perceived stress into a functional adaptation capacity that protects the caregiver’s general health [28,29]. These findings suggest an association between caregiving for individuals with SMDs and varied coping strategies. In this sense, resilience could be related to the caregiving experience, potentially functioning as a modulating factor in the relationship between perceived stress and functional adaptation capacity. However, given the cross-sectional design of this study, no causal or directional conclusions can be drawn from these observations.

From a gender perspective, the overrepresentation of women in this sample highlights the need for interventions that address the specific vulnerabilities of female caregivers. Research has shown that women caregivers experience higher levels of burden and poorer health outcomes compared to their male counterparts, partly due to the intersection of caregiving with other domestic and occupational responsibilities [29]. Public health strategies should therefore incorporate a gender lens, recognizing caregiving as a social determinant of health that disproportionately affects women.

The comparative analysis revealed significant differences in physical functioning, emotional well-being, and bodily pain between caregivers with good versus poor SPH. This finding is consistent with other studies that have documented associations between caregiving burden and physical and emotional health dimensions in caregiver populations. The large effect size observed for bodily pain (Cohen’s d > 0.8) suggests that pain management should be a priority in interventions targeting older caregivers. Chronic pain not only affects physical functioning but also contributes to emotional distress and social isolation, creating a vicious cycle that undermines overall well-being.

This finding is consistent with other studies that have documented associations between caregiving burden and physical and emotional health dimensions in caregiver populations [30]. Institutional actions should prioritize holistic well-being over exclusively disease-centered approaches. This priority is grounded in a reflection on the ethics of care.

The logistic regression analysis revealed that the SF-36 General Health dimension was the only independent predictor of good SPH in the adjusted model. This finding is conceptually consistent, as both SPH and the General Health dimension of the SF-36 capture the individual’s global appraisal of their health status. The General Health subscale asks respondents to rate their overall health and expectations about health changes, which closely mirrors the single-item SPH question used in this study. It is important to note, however, that the conceptual proximity between SPH and the SF-36 General Health dimension—both of which capture the individual’s global health appraisal—may reflect a degree of conceptual collinearity. This overlap may have contributed to the dominance of this predictor in the adjusted model, and the possibility of overfitting cannot be entirely excluded given the sample size. Future studies should consider sensitivity analyses excluding the General Health dimension, or the application of penalized regression methods, to evaluate the robustness of these findings. This integration of the influence of daily experiences on self-perceived well-being extends beyond the biomedical model to include psychological and social dimensions that contribute to positive aging [31]. In this regard, a fundamental challenge for aging research is the recognition that pathophysiological conditions are not the sole determinants of health. Enjoyment and perceived well-being are critical components of quality of life.

The lack of independent association between comorbidity count and SPH in the adjusted model is noteworthy. While comorbidity is an objective measure of disease burden, SPH reflects a more holistic and subjective integration of physical, emotional, and social factors. This finding suggests that interventions focused solely on managing chronic diseases may be insufficient to improve caregivers’ perceived health. Instead, a more comprehensive approach that addresses functional capacity, emotional well-being, and social support may be more effective.

This study has several strengths. First, it focuses on an understudied population—older family caregivers of individuals with SMDs—in a Latin American context where informal caregiving is the norm. Second, it employs validated instruments (Zarit Burden Interview, SF-36) that allow for comparison with other studies. Third, the use of both univariate and multivariate analyses provides a nuanced understanding of the factors associated with SPH.

However, several important limitations must be acknowledged. The small sample size (*n* = 71) significantly constrains the statistical power of this study and increases the risk of Type II errors, whereby true associations may fail to reach statistical significance. The predictor-to-case ratio in the logistic regression model is suboptimal, particularly given the number of candidate predictors examined, which raises concerns about model stability and the potential for overfitting. The limited sample size also precluded subgroup analyses (e.g., by gender, age strata, or burden severity), which could have provided deeper insights into differential patterns of association. Furthermore, the use of convenience sampling from a single specialized center limits the generalizability of these findings beyond this specific clinical context. The cross-sectional design precludes causal inference, the associations found should be interpreted as co-occurrences rather than directional effects, and those associations may be influenced by unmeasured confounders such as social support, economic resources, duration of caregiving, and pre-existing health conditions. The dichotomization of SPH, while consistent with epidemiological practice, may have resulted in loss of information and reduced sensitivity to detect more nuanced patterns of health perception. Additionally, the reliance on self-reported data for comorbidity and other health indicators introduces the possibility of recall bias and social desirability bias. Future research should employ larger, longitudinal designs with probability sampling to confirm and extend these findings, and should consider the inclusion of objective health measures and more granular assessment of SPH.

In summary, the results of this study suggest an association between SPH and the global assessment of health status among older caregivers of relatives with SMDs. These findings are exploratory in nature and should be interpreted within the methodological constraints of the study. From a public health perspective, they may inform the development of policies oriented toward holistic well-being models that consider perceived health as a relevant indicator, while acknowledging the need for confirmation in larger, longitudinal studies.

## 5. Conclusions

This cross-sectional study examined the relationship between self-perceived health and clinical, functional, and burden-related variables in older family caregivers of individuals with severe mental disorders in Bogotá, Colombia. The findings contribute to the limited literature on this vulnerable population in Latin American contexts.

Among the 71 participants, self-perceived health showed an independent association with the general health domain of the SF-36. The count of comorbidities and global caregiver burden did not maintain independent associations after adjustment, which is consistent with the multidimensional nature of health appraisal in this population. These findings suggest—within the limits of this cross-sectional, exploratory study—that SPH may serve as a pragmatic indicator to identify potentially vulnerable older caregivers. Given the small sample size and cross-sectional design, these results should be interpreted as hypothesis-generating, and replication in larger longitudinal studies is warranted.

From a public health perspective, these findings underscore the importance of moving beyond disease-centered models toward holistic approaches that address functional capacity, emotional well-being, and social support. The marked feminization of caregiving observed in this study highlights the urgent need for gender-sensitive policies that recognize and redistribute care work, provide economic support, and ensure access to respite services.

Future research should employ larger, longitudinal designs with a representative sampling to confirm associations and explore potential mediating and moderating factors, and establish the trajectories of health perception over time. Intervention studies are needed to test whether wellness-based programs that target functional and emotional dimensions can improve SPH and overall quality of life among older caregivers of individuals with severe mental disorders.

## Figures and Tables

**Table 1:** Sociodemographic and clinical characterization of the population (*n* = 71).

Variable	Measure (*n* = 71)
Age (Median and IQR)	65 (62–72)
Sex female *n* (%)	66 (92.96%)
Sex male *n* (%)	5 (7.04%)
**Comorbidities**	
Average (Mean and SD)	3.21 (1.90)
**Zarit Caregiver Burden Interview**	
Score (Mean and SD)	48.46 (14.87)
No burden, *n* (%)	37 (52.11%)
Mild burden, *n* (%)	10 (14.08%)
Moderate or intense burden *n* (%)	24 (33.80%)
**SF-36 Dimensions** (Median and IQR)	
Physical Functioning	90 (70–95)
General Health	75 (55–80)
**Good overall SPH**, *n* (%)	50 (70.42%)

IQR: Interquartile Range; SD: Standard Deviation; SPH: Self-Perceived Health.

**Table 2:** Comparison of clinical and functional variables according to Self-Perceived Health (SPH).

Variable	Total (*n* = 71)	Good SPH (*n* = 50)	Poor SPH (*n* = 21)	*p*-Value *	Cohen’s d
**Clinical Characteristics**					
Age (Median, IQR)	65 (62–72)	65 (62–72)	69 (62–74)	0.395	−0.22
Comorbidities (Mean, SD)	3.21 (1.9)	3.02 (1.8)	3.67 (2.1)	0.170	0.36
Zarit Score (Mean, SD)	48.4 (14.8)	46.4 (14.3)	53.3 (15.2)	0.081	−0.46
**SF-36 Dimensions (Median, IQR)**					
Physical Functioning	90 (70–95)	90 (80–100)	70 (45–85)	0.004	−0.89
Role Physical	100 (25–100)	100 (75–100)	50 (0–100)	0.012	−0.88
Role Emotional	100 (33–100)	100 (66–100)	33 (0–100)	0.002	−0.80
Energy/Fatigue	60 (45–75)	65 (55–80)	45 (35–65)	0.003	−0.95
Emotional Well-being	68 (56–80)	72 (64–84)	48 (40–72)	0.008	−0.76
Social Functioning	75 (50–100)	87 (62–100)	62 (37–75)	0.008	−0.61
Bodily Pain	77 (45–100)	89 (67–100)	45 (22–67)	<0.001	−1.12
General Health	75 (55–80)	75 (70–85)	45 (40–60)	<0.001	0.25

SPH, Self-Perceived Health; IQR, Interquartile Range; SD, Standard Deviation; SF-36, 36-Item Short Form Health Survey; Zarit Score, Zarit Caregiver Burden Interview. * *p*-value obtained using the Mann–Whitney U test for continuous variables.

**Table 3:** Factors associated with Good Self-Perceived Health (Logistic Regression Analysis).

Variable	Crude OR	95% CI	*p*-Value	Adjusted OR	95% CI	*p*-Value
**Age**	1.035	0.95–1.12	0.395	1.059	0.96–1.16	0.214
**Sex (Female)**	0.344	0.05–2.29	0.270	0.174	0.01–2.48	0.198
**Comorbidities**	0.824	0.62–1.09	0.172	1.064	0.74–1.52	0.738
**Zarit Score**	0.969	0.93–1.00	0.081	1.010	0.95–1.06	0.685
**SF-36 Dimensions**						
Physical Functioning	1.042	1.01–1.07	0.004	0.992	0.94–1.04	0.741
Emotional Well-being	1.047	1.01–1.08	0.008	1.012	0.96–1.06	0.628
Bodily Pain	1.046	1.02–1.07	<0.001	1.024	0.99–1.05	0.127
General Health	1.101	1.05–1.15	<0.001	1.112	1.05–1.17	<0.001

OR: Odds Ratio; CI: Confidence Interval; SF-36: 36-Item Short Form Health Survey. Significance was set at *p* < 0.05. Hosmer–Lemeshow test: *p* > 0.005.

## Data Availability

The data analysed are contained within a restricted institutional database and cannot be shared publicly or on request due to privacy, confidentiality, and ethical constraints.

## Abbreviations

The following abbreviations are used in this manuscript:

SPH	Self-perceived health
SMD	Severe mental disorder
HRQoL	Health-related quality of life
MNCD	Mayor neurocognitive disorder
OR	Odds ratio
aOR	Adjusted odds ratio
CI	Confidence interval
IQR	Interquartile range
SD	Standard deviation
